# Exploring factors that contribute to human trafficking in Ethiopia: a socio-ecological perspective

**DOI:** 10.1186/s12992-021-00725-0

**Published:** 2021-07-03

**Authors:** Lemma Derseh Gezie, Alemayehu Worku Yalew, Yigzaw Kebede Gete, Florence Samkange-Zeeb

**Affiliations:** 1 Department of Epidemiology and Biostatistics, Institute of Public Health, College of medicine and Health Sciences, University of Gondar, Gondar, Ethiopia; 2 Department of Preventive Medicine, School of Public Health, College of Health Sciences, Addis Ababa University, Addis Ababa, Ethiopia; 3 Leibniz Institute for Prevention Research and Epidemiology – BIPS, Bremen, Germany

**Keywords:** Human trafficking, Exploitation, Social ecological model, Ethiopia

## Abstract

**Background:**

A number of individuals in Ethiopia are involved in illegal types of transnational migration, namely human trafficking and smuggling. The magnitude is not declining despite efforts to curb it. An in-depth understanding of the reasons for trafficking is needed to control human trafficking and its consequences.

**Methods:**

The study included four focus group discussions and 44 in-depth interviews conducted in three border towns, five trafficking-stricken areas, and the IOM transit center for returnees in Addis Ababa. Participants were victims of trafficking, emigrants, community members, police, immigration personnel, and staff of labor and social affairs offices and non-government organizations. Based on the social-ecological model framework, content analysis was implemented using opencode-4.03 software.

**Results:**

Factors identified as possibly encouraging acts of illegal migration included community attitudes such as having a child abroad being considered a measure of status, and the reluctance to do certain types of works associated with low social status when at home. There was, however, willingness to do similar work abroad, which was coupled with the belief that wages for such jobs were better abroad than at home and a lack of knowledge regarding differences in working conditions in the two settings. Further reasons given were poor government service provisions, ineffective immigration policies, political instability, government focus on reactive and poorly coordinated control rather than on sustainable prevention of human trafficking and corruption. Corruption was said to be the underlying factor for the poor coordination among government agencies, communities, and individuals. These factors, among others, led to the loss of trust and belief in local governance, resources, and opportunities to build one’s future at home, thereby creating fertile ground for illegal migration, including smuggling and possibly trafficking.

**Conclusions:**

Individual lack of trust and belief in local governance, resources, and opportunities to build one’s future was the main reason why people resorted to migrate in a manner subjecting them to human trafficking. Thus, comprehensive and coordinated efforts involving government agencies, communities, and other stakeholders are needed to help curb human trafficking and its consequences.

## Background

A healthy transnational migration might be useful for the migrant as well as the source and receiving countries as it might meet the demands of all sides. However, a considerable proportion of global migrants were involved in illegal migration, mainly in human smuggling or trafficking [1]; illegal migration also accounted for two-thirds of all transnational migrants in Ethiopia [2]. Human smuggling is the procurement of the illegal entry of a person into a state party of which the person is not a national or a permanent resident for financial or other material benefits [3]. Human trafficking on the other hand is the recruitment, transportation, harboring or receipt of persons usually by force, coercion, or deception for purposes of exploitation, and can be in the form of labor trafficking, sex trafficking, and organ removal [4]. Thus, the process of human trafficking may follow legal processes and procedures or it can involve smuggling during the movement across borders; however, the relationship between the trafficker and the victim is not sufficiently protective for the latter [3, 4].

Human trafficking, which is becoming an issue of global concern, is affecting Ethiopia significantly [5, 6]. However, little is known about the predisposing factors that subject people to illegal migration in a manner that makes them vulnerable to transnational human trafficking in Ethiopia [7]. Specifically, the mechanism how people from Ethiopia enter into the web of trafficking is unclear [6].

Governments, international agencies, and nongovernment organizations (NGOs) are struggling to combat human trafficking in every aspect. In this regard, the government of Ethiopia took certain measures to prevent trafficking and strengthen anti-trafficking law enforcement. For instance, it organized workshops for the national media to create awareness about trafficking, conducted community conversations, and organized television and radio public service announcements to enhance public awareness, etc. in conjunction with international organizations. The government even banned the recruitment of low-skilled domestic workers for the Middle East until bilateral work agreements were established [5], and recently passed a comprehensive legislation against human trafficking and smuggling [2]. All of these efforts should have significantly prevented innocent people from being deceived or coerced for the purpose of exploitation and the number of individuals transferred illegally should have declined.

However, the magnitude of the problem has rather been increasing over time [2], and because it is a phenomenon that affects the source, transit, and receiving countries [8], it is an issue of international health and human rights violations. On the part of the victims, beyond violations of human rights, human trafficking adversely affects their social wellbeing, which is one indicator of health [9]. Human trafficking also exposes victims to various types of illnesses and injuries. It is now recognized as a major public health problem [10, 11] and Ethiopian victims are not immune to its health consequences [12].

Obviously, combating human trafficking may help in reducing human rights violations, violence, and health and social problems among victims [11]. This is because stopping human trafficking is the same as cutting subsequent problems at their sources [11, 13]. On the other hand, continuing with the current trend of interventions on trafficking has little promise of curbing the practice and its subsequent problems.

The push and pull factors of human trafficking can be examined using the framework of the social-ecological model (SEM). The model has been frequently employed by the Centers for Disease Control and Prevention to study violence [14], a severe form of which is trafficking [11]. In this context, the SEM hypothesizes that human trafficking is the effect of different factors at multiple levels, mainly individual, interpersonal (relationship), community, and societal. Similarly, it is rational to hypothesize that the reasons for human trafficking might not be limited to only individual level characteristics, such as aspects of the victims’ income status, education, or history of violence, etc. Instead, the reasons might also evolve from other higher levels, such as interpersonal (relationship) and community or societal level characteristics that would interact in a complex manner to affect the decisions of individuals to migrate in a way that makes them vulnerable to trafficking [15].

From the higher-level characteristics, the interpersonal (relationship) level could concern social events involving close persons, such as divorce, peer pressure, family conflict, etc. Similarly, the community or societal levels could be about social, cultural, and environmental factors that may include the lack of local job opportunities, drought, or corruption in government institutions [16].

Literature shows that the reasons for human trafficking relating to poverty as push factors affect individuals at all levels of the SEM [16–18]. This is because economic deterioration affects individuals, relationships, communities, and society at large [15]. Poverty encompasses various dimensions, such as lack of access to basic services and insecurity in daily life. In the case of women and girls, it might extend to their disempowerment coupled with poverty that in turn forces poor parents to sell their daughters into domestic servitude, prostitution, or forced marriages [16].

.Different factors at the individual level of the SEM have been identified [19]. Women are often reported to be at a higher risk of trafficking, mainly due to the low value given to them by family, community, and society. Many studies showed that younger people (both male and female) were more vulnerable to trafficking [19–21]. The fact that persons from rural areas lack knowledge or awareness about the realities of trafficking might increase their vulnerability [20]. Illiteracy, limited access to education, and dropping out of school are other individual level factors that have been reported to put individuals at risk of trafficking [21].

At the interpersonal (relationship) level of the SEM, sexual, emotional, and physical abuse as well as neglect [20], peer pressure [22], large family size, and pregnancy outside of marriage which could result in stigma have been reported to be reasons that could drive people into conditions of human trafficking [23–26]. Several studies also pointed out that the influence of deceptive information from brokers, traffickers, and family members about the conditions of work abroad was another reason leading to individuals deciding to migrate [22] in a way that exposes them to exploitation.

Similarly, at the community and societal levels of SEM, socio-cultural characteristics, such as child marriage [8, 27] and traditional dowry practices increase financial burden and force poor parents to sell their daughters [16]. Gender inequality was another reason for human trafficking regarding women as males had more influence than women in some communities, and people still favor sons and view daughters as an economic burden [20, 27, 28]. Poor governance [22], corruption, weakly enforced laws, inconsistent prosecution and application of public policy, fortified organized crime networks, lack of coordination among authorities within governments as well as intergovernmental collaborations [19–21], war, and civil strife [8, 22, 29] were the most frequently reported reasons for human trafficking.

Although some of the factors that drive people into migration have been identified, the mechanism as to how these factors create fertile grounds for trafficking is not clear in the Ethiopian context. Therefore, this study qualitatively explored the reasons for human trafficking in the context of Ethiopia by examining factors relating to individuals, relationships, communities, or societies by using the SEM framework. Each factor from any of these levels of SEM was examined, mainly focusing on the ways it influences the decision-making process of migrants to leave their origins and how it increases their vulnerability to trafficking. Because understanding and acting across all levels of the SEM at the same time might be more successful in sustaining the prevention of trafficking compared to any single intervention, the present study employed the model as its framework for collecting and analyzing the data and discussing findings.

## Methods

### Study design

A phenomenological qualitative study design was employed to explore the reasons for human trafficking from victims’ lived experiences, or victims were encouraged to elaborate on how they got caught up in trafficking. The trafficking status of the respondents was determined based on the United Nations 2000 definition of human trafficking [4] as detailed elsewhere [30–32].

### Study setting

**Fig. 1:**
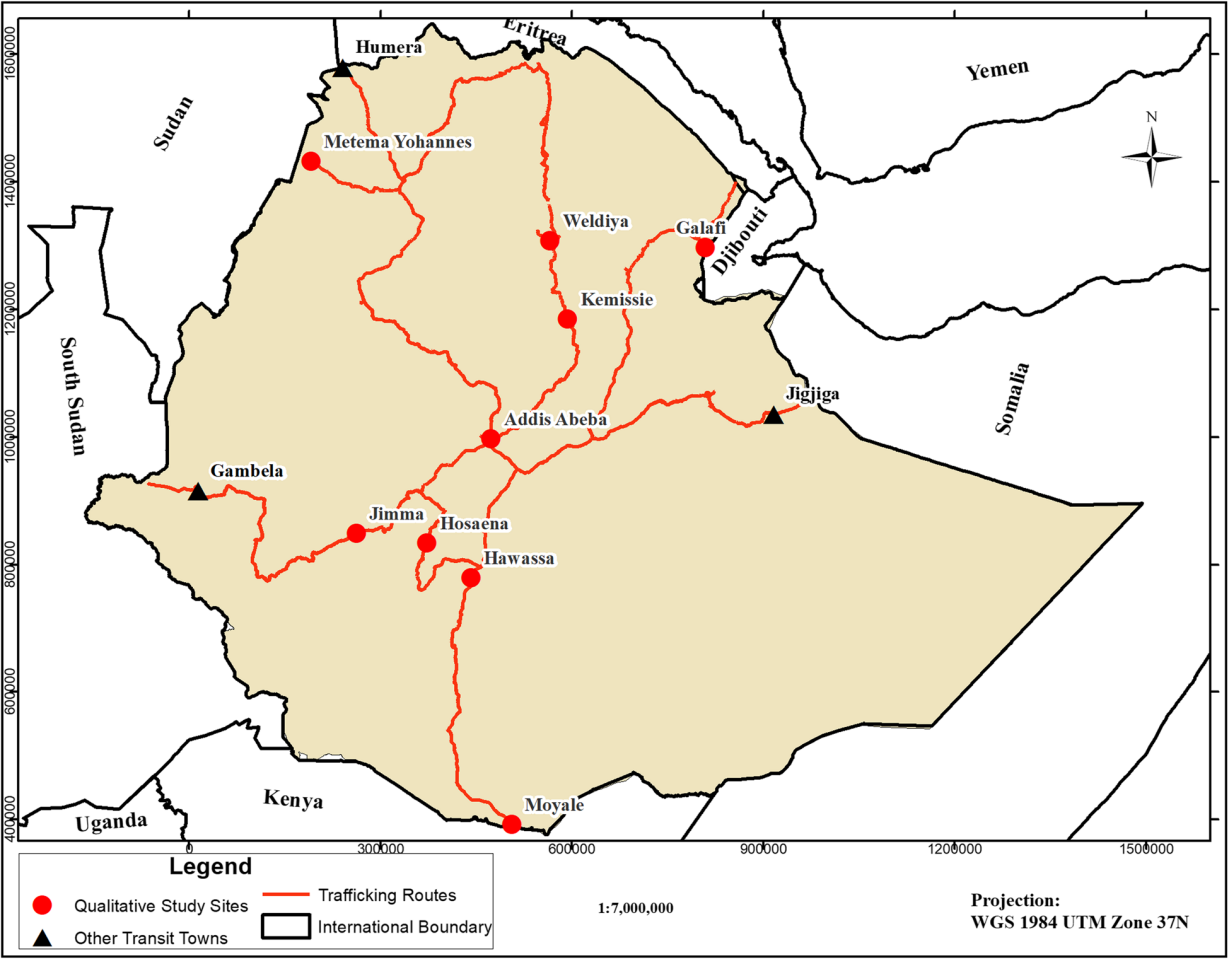
Map of Ethiopia consisting of the nine qualitative study sites

The current study is part of a bigger work focusing on trafficking, sexual violence, and mental illness among irregular migrants, which is discussed elsewhere [30–32]. The study was conducted in three different purposively selected groups of sites in Ethiopia. The first group of sites consisted of five of the areas most affected by human trafficking in the country: Kemissie, Woldia, Jimma, Hawassa, and Hosaena [12]. The second group of sites comprised the three border towns in the three major human trafficking corridors of Ethiopia, namely Mettema-Yohannes, Moyale, and Galafi, which are situated bordering the Sudan, Kenya, and Djibouti, respectively. Mettema-Yohannes and Moyale are located at the points where the Cairo-Cape Town Highway passes through Ethiopia. Galafi is situated on the Ethio-Djibouti Highway and is exactly on the border. The third site is the transit center in Addis Ababa, which is managed by the International Organization for Migration (IOM) and takes care of returnees from different parts of the world on their way to their countries of origin (Table 1). This transit center was selected as a sample site in order to capture the experience of victims who returned by air transport (Fig. 1). The data from the sources were collected between April 2016 and March 2017.

**Table 1:** Research site and type and number of respondents on human trafficking in Ethiopia, 2016

Group of sites	Sites	In-depth interview	FGD
Border towns	Mettemma	8 (4 returnees and 4 from community)	14 (7 police and 7 legal emigrates)
	Moyale	5 (3 returnees and 2 from police)	
	Galafi	5 (3 returnees and 2 from police)	
Areas most affected by human trafficking	Woldiya	6 (3 returnees and 3 from community	
	Kemissie	3 (2 returnees and 1 from community)	
	Jimma	8 (3 returnees and 5 from community)	
	Hawassa	2 (both were returnees)	6 (from community)
	Hosaena	5 (2 returnees and 3 from community)	
IOM transit center for returnees	Addis Ababa	2 (returnees)	7 (returnees)
**Total**		**44**	**27**

### Sampling and recruitment

Victims of human trafficking were recruited into the study on arrival at the three border towns from abroad or at the IOM transit center for returnees from different parts of the world. Newly arrived returnees were approached and informed about the aim of the study, identified some of them who could make an effective interview to share their rich experience with us, and asked if they would be willing to participate in the study in the form of in-depth interviews or focus group discussions (FGD).

To support the information obtained from the victims, other key informants such as emigrants (people leaving Ethiopia legally via the three border towns), employees of respective government organizations (Office of Labor and Social Affairs, the police, and Immigration offices), NGOs (IOM, and Mahibere Hiwot, a local NGO), and persons from trafficking-stricken communities were also included in the study. The aim of studying emigrants was to understand how and why they decided to leave for abroad, and to examine their awareness about the possibility of being trafficked during travel or at destination. The key informants were recruited purposively for in-depth interviews or FGD. They were required to be knowledgeable about the role of social and environmental factors at different levels, such as individual, interpersonal relationships, communities, and societies, on the trafficking process, during recruitment, traveling, destination/exploitation, and integration stages. Participants from trafficking-stricken communities were identified by consulting with local government administrators and staff of government and NGOs working mainly in the field of trafficking or migration. The key informants were then put in four groups composed of the police (in Metemma-Yohannes), victims of trafficking (in IOM transit center for returnees), community members (in Hawassa), and legal emigrants (in Metemma-Yohannes). The aim was to form homogenous discussant groups for each category of key-informants.

### Data collection

During data collection, the FGDs were held at the initial phase of the study, mainly to gain insight into the different perspectives of discussants about the reasons for human trafficking, and to brainstorm ideas on the reasons for human trafficking. The in-depth interviews were conducted in the four groups to understand the reasons for human trafficking by discussing issues in detail, even including the discussants’ personal experiences or observations. As detailed later on in the data processing and analysis section, the number of in-depth interviewees and FGDs were determined based on the saturation of the information gathered [33].

The in-depth interviews and FGDs were conducted in the Amharic language by two interviewers, the principal investigator and a field worker, who both had ample experience in collecting qualitative data. When participants couldn’t speak Amharic, their responses were translated into Amharic by interpreters recruited from the community. To facilitate the data collection process, an open ended interview guideline that mainly helped interviewees to relate how they or other people got involved in the process of human trafficking was prepared. The guideline was built by reviewing literature focusing on reasons for human trafficking. The first section of the guideline briefly described human trafficking, comparing it to the other types of migration, mainly human smuggling. The questions after that focused on individual and interpersonal characteristics that contributed towards the victims’ vulnerability to human trafficking and how each characteristic lead people into exploitation. The guideline also included questions about characteristics at community (including its culture, local governments, etc.) and society levels (e.g. government policies, environment, etc.) among others. Generally, the questions encouraged the participants to discuss the reasons for human trafficking, guided by the SEM.

To facilitate confidentiality and the quality of data as well as to enable sensitive issues to be addressed, the interviews and FGDs were conducted at places where the interviewees felt comfortable. All interviews and FGDs were tape-recorded. During the FGDs, the principal investigator facilitated the discussion, while a colleague made note of particularly striking responses and gestural expressions. To enhance the credibility or internal validity of the study, relevant members of communities who had some connections with the context of human trafficking were recruited to participate. Further, participants were recruited from different sites spread across the country. In addition, efforts were made to develop participant trust in the significance of the study so that open, complete, and truthful responses could be obtained using the appropriate data collection methods. To this end, a considerable period of the study (one year) was dedicated to the collection of data using different methods (in-depth interview and FGD) and different groups of participants were recruited. The interview was performed strictly following the World Health Organization ethics protocol recommended for interviewing trafficked women so as not to re-traumatize them [34]. During all these activities, data obtained from participants were kept anonymous and confidential. The length of the in-depth interviews ranged from 27 to 48 min and of the FGDs from 53 to 72 min.

### Data processing and analysis

The analysis of the qualitative data was started parallel to the data collection process as successive probing questions were formed based on the participants’ responses. The collected data were transcribed and translated into English and then coded and organized thematically using the software opencode-4.03. The data were examined using content analysis, whereby each phrase or statement was coded according to the concept it contained. The SEM framework was then employed as a guiding theoretical framework at the individual, interpersonal, and community/societal levels. To this end, the codes, respectively emerging themes, were grouped or summarized and tabulated for each of the three levels or their combinations. To help the reader better understand why and how people were being trafficked, and also who could be at risk of being trafficked, the groupings of the themes are supported with examples of direct (verbatim) quotations as shown in the results section.

To maintain the dependability of the study, all procedures, findings, and decisions passed were documented. Furthermore, the qualitative data were shared with colleagues so as to get peer-feedbacks and check the confirmability (neutrality) of the analysis and its interpretations.

## Results

### Background characteristics of participants

The current study was based on a total of four FGDs and 44 in-depth interviews. Two of the FGDs were conducted at Metemma Yohannes (one comprising police officers and the other legal emigrants). The third FGD was held in Hawassa city and was made up of community members, while the fourth, which comprised victims of trafficking, was conducted in the IOM transit center for migrants. The FGD conducted in Hawassa was made up of six persons and the remaining three of seven persons each (Table 1). Fourteen of the in-depth interview participants were from Oromiya, eleven from Amhara, ten from Southern Nations Nationalities and Peoples Regional State (SNNPRS), five from Tigray regions and four from Addis Ababa. Further, the interviewees were aged 14 to 47 years and slightly less than half of them were male (*n* = 21) (Table 2).

**Table 2:** Socio-demographic characteristics of interviewees and discussants on human trafficking in Ethiopia, 2016

Characteristics	In-depth Interview Number (percent)	FGD Number (percent)
**Age (in years)**		
14–17	3 (6.8)	0 (0.0)
18–25	11 (25.0)	9 (33.3)
26–35	16 (36.4)	11 (40.7)
36–47	14 (31.82)	7 (25.9)
**Educational level**		
Primary	8 (18.2)	0 (0.0)
Junior	10 (22.7)	7 (25.9)
High school	18 (40.9)	13 (48.1)
Certificate and above	8 (18.2)	7 (25.9)
Not reported	0 (0.0)	
**Gender**		
Male	21 (47.7)	18 (66.67)
Female	23 (52.2)	9 (33.3)
**Marital status**		
Never married	12 (27.3)	4 (14.8)
Married	19 (43.2)	15 (55.6)
Separated	8 (18.2)	5 (18.5)
Widowed	5 (11.4)	1 (3.7)
Not reported	0 (0.0)	2 (7.4)
**Region**		
Oromyia	14 (31.2)	7 (25.9)
Amhara	11 (25.0)	11 (40.7)
SNNPRS	10 (22.7)	6 (22.2)
Tigray	5 (11.4)	1 (3.7)
Addis Ababa	4 (9.0)	1 (3.7)
Others	0 (0.0)	1 (3.7)
**Religion**		
Muslim	19 (43.2)	8 (29.6)
Orthodox	13 (29.5)	12 (44.4)
Protestant	10 (22.7)	7 (25.9)
Others	2 (4.5)	0 (0.0)
Not reported	0 (0.0)	2 (7.4)

### Reasons for human trafficking

Based on the SEM framework, the characteristics that lead to human trafficking that were identified from the in-depth interviews and FGDs (Table 3) were summarized at four levels. To begin with, characteristics of individuals given as the reasons for trafficking were classified as individual level (Theme I), while reasons for trafficking that were related to the relationships of the victims to persons close to them were classified at the next level of the model, the interpersonal/relationship level (Theme II). Characteristics of communities or societies that drive people into human trafficking were classified at the community or societal level (Theme III). Finally, a fourth level, under which factors that appeared at all of the three levels of the SEM mentioned was created (Theme IV). The corresponding factors were poverty and loss of trust and belief in local governance, resources and opportunities to build future welfare by working at home. The (sub-) themes that emerged under each of the four levels are summarized hereafter.

**Table 3:** Codes and Themes as per applied content analysis based on the SEM framework

No	Synthesis (Themes)	Code
1	**Individual level**	
	➢ Socio-demographic and economic factors	
	• Age related aspects	✓ Emotional decision, risk-taking behavior, unaware of brokers conspiracy
	• Gender-related aspects	✓ Nature of the work that restricts their movement (e.g., housemaid), unable to self-defend physical attacks for biological or other reasons
	• Residential area-related aspects	✓ Inaccessibility to information (by internet, Media, etc.), ✓ Inaccessibility to offices that process immigration requirements ✓ Undermining traveling hardships ✓ Considering air travel as a luxury
	• Financial resources-related aspects	✓ Unable to offer for safest travel and processing expenses
	➢ Smuggling status	✓ Being smuggled, use of employment agencies, having contractual agreement with employers before departure
2	**Interpersonal/relationship level**	
	➢ Events on family	✓ Divorce and family conflict
	➢ Close relation with colleagues abroad	✓ Considering colleagues abroad as their only role model/reference for future success (pull factor) ✓ Frequent communication with colleagues abroad (pull factor) ✓ Peer pressure from abroad (pull factor)
	➢ Pressure from other/s	✓ Pressure from family, colleagues at home, brokers, etc.
3	**Community/societal level**	
	➢ Poor community attitude on what it has ➢ Poor government service provisions ➢ Focus on controlling than preventing trafficking ➢ Poor coordination in anti-trafficking efforts ➢ Political conflict ➢ Corruption ➢ Environmental Problems	✓ Considering immigrants working abroad as heroes ✓ Being inhospitable for returnees and deported persons ✓ Illogically thinking that traveling hazards are predetermined fates for each individual ✓ Degrading some types of jobs at home and attaching low social status to them, and willing to be engaged in same jobs abroad ✓ Misunderstanding about returns from foreign jobs without the consideration of working hours allocated in the two settings ✓ Unable to get work place, lengthy bureaucratic process, unable to get farm land (mainly for youths) ✓ Focusing on border control, poor (or only campion based) intervention or awareness creation efforts on the community ✓ Poor government system in fighting trafficking, less cooperation from the community, poor law enforcement of traffickers, etc. ✓ Unsafe migration (to escape from arrest) after involvement in road side violence against government ✓ Unfair treatment of citizens (favoring for relatives or to whom bribe officials by money, etc.), releasing brokers arrested, etc. ✓ Draught and poor crop production
4	**Raised at all levels of SEM**	
	• Lack of trust and belief in building future welfare in home-country	✓ Perceived no hope by working at home-country ✓ Loss of trust and belief in home country opportunities ✓ Perceived oversea life is the only way-out for better life ✓ Attitude of having a relative abroad as a measure of status
	• Poverty	✓ Narrow option for livelihood, opportunities, or basic services ✓ Mistreatment as a human being and freedom of expression

### Theme I: individual level characteristics

The following individual level characteristics were identified as increasing the risk of individuals to follow unsafe modes of migration that could lead them into trafficking or force them into working conditions that could expose them to traffickers: female gender, young age, residing in a rural area, originating from a poor family, and being a smuggled person; poverty and loss of trust and belief in local governance, resources and opportunities to the possibility of leading a modest life at home were two important factors that also appeared at the individual and other levels of SEM. The reasons why these groups of people were more vulnerable to trafficking than their counterparts are discussed under the following two sub-themes (socio-economic characteristics and smuggling status) **as** well as in the fourth theme.

### Socio-economic characteristics

#### Gender-related aspects

Women and girls are usually employed as domestic workers, work that subjects them to restricted freedom of movement and generally puts them at risk of exploitation, which is a major sign of trafficking. On the other hand, as men’s and boys’ jobs are usually outside of the home, they are not affected by this type of exploitation. Understanding this reality, traffickers usually sell females to series of networked traffickers. As the number of successive buyers increases, the amount of money the trafficked women and girls must pay to free themselves from the traffickers also increases. They, therefore, fall under debt bondage that must be paid from the salary they earn by working in households. “*When I left my family, I paid Br. 300 to an Ethiopian broker. When we reached Metemma, the Sudanese brokers took us through the desert mostly on foot. … When we reached Khartoum, another Ethiopian broker who bought us from the Sudanese brokers took us to his house … After two days, he delivered us to our employers who paid our salary to him … He took my salary for eight months. When I started speaking Arabic, I escaped from him …*” (Interviewee #16, woman).

Moreover, due to the nature of their jobs, women and girls are subjected to other types of exploitations, mainly extra working hours. “*… The extra working hours were not only in the household that I was employed in Saudi, but also after finishing all the work in my employer’s house, I would be forced to do the same work for her mother, sisters, aunts, and in any other relatives’ houses … Strict control prevented me from escaping; mothers or someone would be assigned as watchdogs. When they wanted me to work in any relatives’ house, my employer herself would take me to the houses, and I would be delivered to them or she might stay there until I finished the work …”* (Interviewee #29, woman).

Men and boys on the other hand are usually engaged in different types of duties that allow them to meet with different persons. This fact might help them find less exploitive jobs. However, this doesn’t mean that they are free from exploitation. “*I was employed as a driver in a household just to take children to and from school and for shopping purposes. However, I was ordered by each member of the household to do other additional tasks. Sometimes, I worked for their relatives too …*. *In short, I worked the whole day and most of the night … The good thing was that when I moved from one place to another, I could have a chance to look for other less exploitive jobs.*” (Interviewee #14, man).

#### Age-related aspects

Participants repeatedly reported that younger migrants were more at risk of being deceived and convinced by brokers or peers to be recruited and coerced by traffickers than older ones, mainly due to their risk-taking behavior. A male participant, who at the time of departure was 13 years old, said “*I left my origin believing what my broker and colleagues told me about the situations abroad. However, upon arrival things were different. The working hours, the difficult levels of tasks, and the burden, in general, were beyond the limits of what is acceptable. I was unable to complain to my employers as I was not mature enough to do that …*. ”.

#### Residential area-related aspects

Key informants also reported that people from urban areas effectively used their access to information and proximity to government offices that processed immigration documents to ensure their safety and reduce the chances of deception and exploitation during recruitment, travel, and at destination. Moreover, migrants from urban areas were mostly from educated families with better financial capacity. This enables them to buy air tickets or choose other safer ways to avoid illegal travel and subsequent hardship as well as exploitation. Persons from rural areas on the other hand were more likely to have to resort to traveling on foot through deserts and facing many hazardous conditions. This also generally entails starting off illegally and usually results in trafficking. *“… Usually, people from urban areas are aware of the difficulties of illegal migration and their proximity and accessibility to immigration offices would also facilitate their safe migration. Moreover, their parents could pay for their air ticket, which would be considered as a luxury for rural migrants who usually opt for traveling on foot … , and all these conditions could expose rural migrants to exploiters and robbers*” (Interviewee #38, man).

#### Financial resources-related aspects

As has already been indicated, due to their illegal status, migrants from poor families travel through unsafe routes and conditions that are likely to expose them to dangerous conditions, including death from diseases, thirst, attacks from dangerous animals, as well as drowning. They are also likely to get caught up in the extended network of brokers who torture or force them to bring money from their families and handover to perpetrators, or recruit them for military purposes in foreign forces or to work under dangerous working condition etc. *“… As migrants from poor families can’t t afford air tickets and expenses related to immigration processes, they usually opt for unsafe routes that usually expose them to traffickers ….*” (Interviewee #35, woman).

However, although having a better economic capacity could prevent people from the risky ways of migration, such as smuggling, it was not a guarantee for reducing migration. In this context, one man said, *“… There are people who went abroad with a huge amount of money in their hands. Last time, my colleague in Adeiago (part of Woldiya) was planning to go to the Middle East as he was unable to start a business with his Br. one million (US$ 44,660.00) …”* (Interviewee #5, man).

### Smuggling status

The present study showed that the relationship between smuggler and smuggled persons is usually better at the beginning of the travels, but changes for the worse during the travels. This happens as the smugglers gain control over the smuggled persons and the latter are sold by a series of brokers or smugglers to traffickers. The smuggled persons are tortured and forced to pay amounts of money that are higher than the amount their previous smuggler would have paid for them. The money has to be sent by the smuggled persons’ families back home or elsewhere. Alternatively, the traffickers would force them to work for certain employers and take their salaries on a regular basis. Traffickers in Yemen were reported to at times even contact the victims’ families and threaten to remove the victims’ organs to compensate for their expenses unless the requested sum of money was paid. By using either of these means, smuggled persons sometimes end up being trafficked.

*“… After sailing through the Red Sea, we reached Yemen and then entered a hidden camp that was guarded by armed traffickers. Then, they grouped us by our ethnicity as Amhara, Oromo, and Tigrie, etc. Torturers who could speak the respective languages of each ethnic group were assigned to beat us …. I joined the Oromo group and all of us were ordered to give them the telephone numbers of our relatives who lived in countries other than Ethiopia, mostly in Saudi so they [our relatives] could send huge amounts of money to their account. The first person was severely tortured, and we were all terrorized by what they did … Most of us paid money ranging from Br.10,000 to 65,000 (US$ 446.00 to 2,900.00) ….”* (Interviewee #1: man)

On the one hand, it was reported that once smuggled persons reached their destination countries free of trafficker control, they were less likely to be exposed to further exploitation, especially in the Middle East. Unlike legal migrants, smuggled persons would freely change their workplaces, looking for better payment or comfort as they wouldn’t have any signed agreements legally binding them to their employers. *“… Once you entered the Middle East illegally, things might be better. As far as you are a hard worker and not engaged in illegal activities, like drug trafficking, you can get better payment without any exploitation ….”* (Interviewee #4, man).

Non-smuggled or legal migrants on the other hand have to go to the countries of their employers after they sign contractual agreements that have to be adhered to, and this condition usually subjects them to continual exploitation. In this context, a non-smuggled woman said, “*My family paid all the necessary expenses for me, and I went to one of the Middle East countries signing a contractual agreement with my employer. Upon arrival, they took my passport and thereafter considered me just as their own property. Unless I finished all the work they ordered me to do, they warned me that they would deport me. …”* (Interviewee #15, woman).

Key-informants however, disclosed that the new Saudi policy of expelling all illegal immigrants could change the condition that favored smuggled persons with regards to exploitation or trafficking.

### Theme II: interpersonal level characteristics

The present study showed that divorce and family conflict were some of the reasons for migration. Moreover, most migrants usually consider their colleagues who are working abroad as their only role models or references for their future life. In addition, the colleagues who are living abroad usually oversimplify the travel hazards as well as exploitations at destination and encourage them to be smuggled.

Some families, colleagues, and acquaintances motivate especially the youth to start traveling abroad, even illegally. A discussant reported, “*Surprisingly, some parents mention few individuals by name, especially those who were previously in their neighborhood but are successful in their life after migration and ask their children why they are not migrating as those individuals rather than living in poverty here …”* (FGD #2: participant #3, woman).

All this serves to create fertile ground for brokers to recruit the young, exercise control over them, and pass them on to traffickers for further exploitation.

### Theme III: community or societal level characteristics

A number of factors at the community or societal level that interfere with the rights and interests of many citizens regarding working at home were identified. For instance, it was observed that some negative attitudes and beliefs that could encourage irregular migration or create inhospitable conditions for the youth had developed within the community. These are discussed hereafter.

### People in communities or societies

According to FGD participants and in-depth interviewees, there is a general awareness of the bad outcomes of trafficking and smuggling within the community/society. However, what is uppermost in people’s minds is only a future involving an improved life. The people believe that the bad health outcomes, deportation, exploitations, and inhumane experiences of migrants were mainly due to their (the migrants’) own wrong decisions, such as following a wrong route and poor communication with employers or security forces along the way. Misfortune is also misunderstood as bad luck or a predetermined fate happening only to few persons. Thus, people in the communities do not give due attention to hazardous events and exploitations that could happen to them during travel and at destinations, unless they experienced them personally. “*I had full information about exploitations and the hazards, like hunger, thirst, forced sex, etc., before my departure … I thought that it was up to the fate of each of us and those worse conditions would happen to only few unlucky migrants … However, the learning came on later when I faced similar challenges during my journey …”* (Interviewee #17, woman).

Another aspect that evolved concerns the negative attitude of communities towards returnees and deportees. According to the interviewees, communities do not offer respect and hospitality to returnees and deported persons as they normally comprise a group of migrants who will have failed to reach their destinations and hence could not start helping their parents financially. Further, parents of returnees were also said to have lesser standing in the community compared to parents with a child working abroad.

Migrants who reach their destinations are said to be perceived to be heroes by the communities back home, while deported children are treated negatively by their parents, who do not feel comfortable having them around. These factors serve as push factors for the returnees that subject them to another round of migration. Similarly, such negative attitudes force others who are not doing well in life at their birthplaces to decide to embark on risky journeys that could expose them to traffickers.

“*I made two unsuccessful attempts to go to South Africa during the last three years and was deported twice … I couldn’t lead my life here in Ethiopia because the community and my parents are not hospitable to me … Even elders and church leaders couldn’t reconcile me with my parents. …. Now, I am working in this small shop just to collect money for a third attempt …”* (Interviewee #25: man).

Key-informants also disclosed the migrants’ tendency to compare the different types of jobs that people look down upon back home with what they usually do abroad, as well as the amount of time people spend working here and abroad. “*Here we are degrading some jobs like caretaker, gardening, housemaid, working in informal businesses, etc. those of course are what we do in other countries for 20 or more hours a day. … If one works on cobblestone for 16 hours here, surely the return will be much better than what could be obtained abroad …”* (Interviewee #8, woman).

The misconceptions described were guiding people to decide on selecting unsafe travel modes including smuggling that sometimes ends with trafficking.

Participants also pointed out that persons doing low-skill jobs or running informal businesses are not treated with respect by their communities of origins. This was said to be discouraging and had made them decide to go abroad to work in the low-skill jobs people in Ethiopia usually associate with lower social status. The attitude towards such jobs is supposed to be different elsewhere. *“… I was giving tea and coffee services over the roadsides of Khartoum. The poor and the rich as well as people in power and low-level workers had services altogether in that open air; their attitude was encouraging. This was not common in the community I grew up in Ethiopia …”* (Interviewee #20, woman).

According to the discussants, the participation of communities in fighting human trafficking is usually poor and depends upon the commitment and determination of the government. A policeman said, “*If people see us working on it seriously, they cooperate with us; otherwise, they don’t.”* (FGD #1: participant #5, man)*.*

On the other hand, another informant said, *“While we were going abroad illegally through the Afar Region, people in the community were seeing us and were aware of our aim but were not vigilant. They neither advised us to cancel our journey nor reported to the police to stop us.*” (Interviewee #9, man).

### Government agencies and policies

Key-informants recalled that over the last few years, the Ethiopian Government had formulated policies and regulations to curb the problem of human trafficking and that some measures had been taken. For instance, when brokers were arrested, attempts were made to take them to court. These attempts were however said to be insufficient. For example, when smuggled persons were arrested at the border, the responses from the respective bodies and offices regarding ensuring the return of migrants to their places of origin and tracing the brokers and taking them to court were said to be poor.

These and other similar challenges or limitations in the anti-trafficking processes that were raised during the in-depth interviews and the FGDs are summarized under the following sub-themes presented hereafter: poor integration among various concerned government offices, corruption, poor government service provisions, and inefficient immigration policies.

### Poor coordination among concerned government offices

Participants had doubts about the strength and reliability of the system established to return victims of trafficking to their places of origin. An informant exemplified his reservation saying, *“… As we do not get confirmation letters from higher-level government organizations that receive victims of trafficking arrested at the border, we cannot say with confidence that the victims reach their origins. … This is because we had the experience of re-arresting smuggled persons that we had already delivered to a government organization …”* (Interviewee #23: man).

Similar poor coordination between government offices was reported when it comes to preventing irregular migration and the reintegration of returnees.

### Corruption

The main challenge faced by anti-trafficking initiatives that was identified during the study was corruption which might affect negatively the capacity of the government to implement policy measures. For instance, a policeman said, “*Last time we arrested 21 smuggled Ethiopians who came from Region X. We had information that those people had already been sold for more than Br. 200,000 (US$ 8,932.00) and this would make the processes of returning them more complex because of corruption … We took them to government office Y to return them home. However, they were released due to lack of government budget to transport them to their origin and resumed the smuggling process … There were times that higher government bodies shouted at us saying, ‘Who ordered you to arrest these people?*” (Interviewee #23: man).

A woman from a government office explained the challenges faced in the process of prosecuting brokers as follows, *“… I never see a heavy measure being taken on a broker. If brokers are accused, they know that they will be released by paying money for bail which can be compensated from one victim …. Because the witnesses and policemen who arrested the criminals did not get protection, they could not give their eye witness accounts properly, and sometimes the courts delay the processes so that the witnesses are not able to present themselves at courts … When a policeman’s efforts are made fruitless, then he feels hopeless and starts to share the benefits of the evil with brokers, or gets corrupted ….”* (Interviewee #2: woman).

### Poor government service provisions

Several key-informants reported that in addition to the corrupted system in some offices, the government had failed to provide new jobs and opportunities for the youth. The situation was said to be discouraging due to the lengthy bureaucratic processes encountered when trying to get a job or even to start their own businesses. According to the key-informants, these were typical features of poor governance. A key-informant who was a barber at the time of the interview said, *“… If two persons try to enter a new business in Ethiopia, the business- making playing field will not be fair for both of them. … Unless you have a relative who is in power or you have money to pay and bribe them …*. , *you cannot work in your own country …”* (Interviewee #3, man).

The key-informants also disclosed that the fact that some government services did not encourage or support entrepreneurship was another push factor for migration. The barber cited in the foregoing paragraph expressed his feelings saying, “*Look at the problem of electric power even! Now it is off for the sixth time since this morning. How can I support my family if they are cutting the power more than six times within six hours? …”* (Interviewee #6, man).

### Inefficient immigration policies

As from 2013, the Ethiopian Government banned the legal travel of migrants looking for low-skill jobs in the Middle East. However, as it is still possible to get visas for countries like the Sudan, some people have been leaving Ethiopia legally and traveling to Sudan, from where they then proceed to the Middle East illegally. One can therefore not be certain whether this immigration policy had the desired effect, that is, to reduce the magnitude of irregular migration and the associated life-threatening incidents, including exploitation.

One key-informant raised his concern regarding this seemingly contradictory immigration policy and asked, “*Why was the direct and safest journey from Addis Ababa to the Middle East closed if we knew that people who moved legally through the widely opened gate (Sudan) would be smuggled into the Middle East? This condition is exposing migrants to death in seas, at the hands of terrorists, and to exploitation by brokers or tortures …”* (FGD #3: Participant #6, man).

There was general consensus among the key-informants that the strategies designed by the government and some NGOs focused more on controlling rather than preventing human trafficking. It was argued that sustainable interventions that would help to change youth attitudes towards human trafficking were not being implemented at grassroots levels. Instead, the focus was said to be on arresting persons who were already trafficked at border districts. Existing prevention measures were reported to be conducted only in the form of campaigns.

Criticism was also raised against the media, which was said to react only when there was something bad to report about the migrants, for instance, when they had drowned in the sea, instead of continually working on the topic of illegal migration to help bring about attitudinal changes. *“… Unless efforts are made to change the attitude of potential migrants at their places of origin, we can’t bring sustainable change here at the border …”* (Interviewee #23: man).

### Political instability

The recent political instabilities witnessed in large parts of the country also served to increase the magnitude of migration. Attempts to escape from government arrests after violence in the streets had forced individuals to leave their places of origins under unsafe conditions that sometimes resulted in recruitment for slavery or exploitation in Libya and Yemen, and/or causalities at sea.

### Environmental characteristics

Both key-informants and in-depth interview participants emphasized the role played by environmental factors. In this regard, land degradation, shortage, or absence of farmland, especially for young persons, as well as frequent drought episodes affecting large areas of the country were said to lead to deterioration in crop production, thereby compromising the capability of families to sustain their livelihood. These environmental forces were reported to be major driving forces for unsafe migration of rural persons that make them vulnerable to human trafficking.

### Theme IV: characteristics at all levels of SEM

Two push factors were observed to be present at all levels of the SEM, namely poverty and gross preference of foreign life or loss of trust and belief in local governance, resources, and opportunities to lead a modest life at home. In the context of our study, trust indicates the confidence individuals have in opportunities, resources, and good governance in their home country, compared to in their potential destination countries. From the statements made by the study participants, it appears that the strong and long-lived cultures and beliefs of considering one’s origins as the preferred living and working places have changed over time. This, coupled with adverse economic conditions, was said to have affected interpersonal relationships (e.g. couple or family), communities or societies at large, making individuals vulnerable to human trafficking.

In this regard, key-informants discussed the cultural beliefs of the majority of the communities on migration. There was general agreement that in various communities across the country, parents consider sending children abroad as a measure of status due to misunderstandings or the poverty that exists at individual levels or within communities or societies. According to one of the key-informants, *“... Sending children abroad is considered as a guarantee to welfare here in the lowland area of ‘province X,’ and usually families compete with each other regarding the number of children they have abroad … Students discuss at school how they will go to Arab countries, not about their education … There are also situations where parents force their children to go abroad ….”* (Interviewee #19, man).

Further, almost all key-informants reported that many people did not trust and believe in government and provision of opportunities that they can improve their future life by working at home. This attitude was said to be present not only at the individual level, but also to be shared by individuals with those closest to them, as well as within families or even communities. This negative attitude that exists in most families or communities was emphasized by one key-informant who said, “*There is an attitudinal problem among us. We do not believe that one can become wealthy by working at home ….”* (FGD #2, participant #3, woman).

As a result, some individuals believe that their fate is dependent upon whether or not they were able to get into one of their preferred destinations under difficult situations that increase their vulnerability to trafficking.

A negative attitude and lack of belief towards the possibility of developing at the place of origin by starting from a very small business and working up to a larger one was also reported to prevail among some community members. The affected persons were said to be some youth and their parents who do not appreciate what their country has to offer, but instead, believe that once they go abroad, they can become wealthy in a short period of time. In this context, one woman stated her feeling saying, *“To grow from a small business to a larger one is unlikely in the minds of some Habesha people. We don’t have trust in what we have; we simply appreciate others’ …”* (Interviewee #24, woman).

## Discussion

In the current study, whose aim was to investigate reasons for human trafficking in Ethiopia and how these influence the decision-making process of migrants, various factors ranging from individual to community or societal level characteristics were identified. Among others, the factors included young age, female gender, low family income, adverse environmental factors, and community attitude towards foreign life, in particular, the opportunities and values attached to it.

During discussions with key-informants on existing efforts to prevent and control human trafficking, the following societal factors were brought up as being detrimental: corruption, inadequate government commitment and determination to stop human trafficking, lack of coordination between government institutions, and inadequate immigration policies. The contextual boundaries of these findings and the reasons for human trafficking were identified and clearly indicated at different levels of the SEM that included individuals, interpersonal relationships, and communities or societies thereby enhancing their transferability.

### Individual factors

The fact that younger people were reported to be more vulnerable to human trafficking than older ones is not surprising as they are bound to have little prior information on the risks of illegal travel. They are also more likely to fall into the hands of brokers and exploiters and be easily deceived by them. Due to their young age, they can also be sold to traffickers by their ‘care givers’ more easily than older persons. Similar findings regarding young age were reported in a systematic review focusing on human trafficking in Southeast Asia [19] and a study conducted in the Philippines [20].

Although the aspects of gender inequalities and disparities at home did not come up in our study, they have been reported to increase the vulnerability of women and girls to trafficking [32]. Further, the nature of work mainly accessible to women, domestic work, places them at risk of exploitation through traffickers as well as employers. Our findings on this aspect are in line with those of previous studies that reported on how those working as domestic workers had limited freedom of movement and hardly any contact to persons outside the household they lived in [6, 29].

Another important aspect that emerged from the study is the susceptibility of rural dwellers to trafficking due to lack of information regarding traveling and destination conditions and poverty. Travel information, including on the dangers of illegal traveling and risks of human trafficking, is more easily accessible in urban areas, where relevant government institutions are based. Regarding poverty, the detrimental environmental changes that have been observed in different parts of the country again mostly affect rural dwellers, who rely on crop production and livestock for their livelihood. The difficulties rural dwellers face might lead them into being lured by traffickers and smugglers with the promise of fast journeys and work at their destination [29, 35].

Poverty, however, does not only force rural dwellers into illegal migration, but also urban dwellers. For instance, according to a report published by the Worker Rights Consortium in December 2018, workers at one of the growing industries in urban areas in Ethiopia, the garment industry, are reported to earn the lowest wages the organization had recently documented in any garment exporting country [36]. The salary of less than 25$US does not enable the workers to lead a decent life, which puts them and their families at higher risk of human trafficking [21]. A United Nations Development Programme study on women in manufacturing in Ethiopia showed that women tended to be over-represented in labor-intensive and low-skilled jobs, also in the garment industry [37]. The prospect of a better-paying job elsewhere is therefore bound to be appealing for many people living in poverty. Although there is no exact data to substantiate this, there are reports that a high number of Ethiopian women and girls are trafficked to the Middle East, where they are employed as domestic workers [21, 38].

### Relationship, community, or societal level factors

The fact that in our study not only those lacking financial resources resorted to illegal migration, but also those who were considerably well-off indicates the presence of other underlying causes. The bureaucratic and unfriendly procedures job-seekers, as well as entrepreneurs, have to go through are bound to have led to the loss of trust and belief in government to work and lead a modest life in one’s own country, which was reported in our study. A logical consequence is the worshipping of foreign countries, evidenced by the preference of the youth to go abroad and having children abroad becoming a status symbol for parents in some communities. The desperation to go abroad makes it easy for traffickers and smugglers to take advantage of the people and some of them end up being caught up in the web of traffickers and brokers, with little chance of escape [35].

Further, the importance attached to having a family member abroad has led to shunning of returnees or deported persons by some communities and families, who view them as failures. This rejection is what at times forces some people to rejoin the cycle of illegal migration, despite the dangers involved.

The way communities look down upon certain types of jobs, for example, cleaning, gardening, and caretaking might indeed discourage young people from taking up such jobs in their own country. This attitude is however probably a result of the expectations families have for the future of their children after having invested in their education. The young are also bound to have high aspirations for themselves, which increase with the level of education attained [39] The change in aspirations, respectively the disparity between the aspirations and what the local situation can offer, have been said to be significant forces that shape migration towards urban areas or abroad [40]. The fact that in our study people were reported to be willing to do the same type of jobs looked down upon back home when abroad to some extent contradicts the argument of aspirations and expectations. It is however known that many people from low-income countries earn more abroad than at home, even when they have low-skilled jobs abroad. Further, the stigma attached to the jobs at home is not necessarily evident when abroad. However, as has been reported in other studies, the downside of such jobs is that they expose the migrants to exploitation, particularly regarding the long hours they have to work per day [6, 35]. Further, the cost of living abroad is generally higher than at home, which leads to many people having to work very long hours or have more than one job for them to be able to sustain themselves and still send money back home [29].

Most of the reasons for human trafficking identified in the current study are factors that interactively function to diminish the trust and belief of individuals and communities or societies in their homeland as a preferred place of work. As has already been discussed, this serves as a strong push factor that drives people to embark on journeys that increase individual vulnerability to trafficking. For instance, miscalculations of benefits that can be obtained by working abroad and the existing culture of ranking jobs and attaching certain social status to people who are engaged in those jobs could influence the trust and belief in local governance, encouraging migration. The exaggerated positive advertisement of foreign opportunities by brokers, colleagues, or families [35] may also reduce the interest of individuals to work at home. Other factors that have already been discussed that negatively affect trust and belief in one’s own home country are corruption, poor government service provisions, political instability, and unfair treatment of citizens for available opportunities [6]. Poor crop production due to environmental degradation and little or no farmland, especially for the youth, also play a role in this respect. Last but not least, the negative attitude of the community towards returnees and deportees and the consideration of having a relative abroad as a measure of status might also lead to a loss of trust and belief in working at home [6, 29].

Our findings regarding the ineffectiveness of attempts to stop trafficking by arresting trafficked persons as they cross borders indicate the need for human trafficking to be considered as a public health problem requiring public health approaches to address it [11, 41, 42]. The fact that poverty and loss of trust and belief in working at home were observed to increase the vulnerability of individuals to human trafficking at all levels of the SEM points to the need for measures that modify social, behavioral or attitudinal, and environmental factors at different levels [15, 41]. Importantly, interventions need to be geared towards reducing individual vulnerability to human trafficking, mainly by building the welfare of individuals, communities, and societies through the provision of employment opportunities and making conditions conducive for self-employment; if such measures are taken, the magnitude of irregular migrations can be minimized, and thereby human trafficking can be reduced [7]. In addition to the public health approach which requires research-based intervention to prevent the problem, reactive measures such as stricter border controls could help stop the problem of human trafficking. However, this also requires intergovernmental collaborations, the coordination of different government agencies, communities, NGOs and other stakeholders [43], as well as putting an end to the effects of corruption that hinder the reintegration of migrants and the sentencing of traffickers and brokers [18].

## Conclusions

The socio-demographic and economic characteristics of individuals, including aspects of young age, female gender, rural residence, and low family income were some of the reasons reported to contribute towards the vulnerability of individuals to human trafficking. The culture of degrading some types of jobs and according individuals a certain social status based on the type of work they do, coupled with the belief of high financial gains when working in foreign countries also increased susceptibility to human trafficking. Other important factors said to push individuals towards (irregular) migration, placing them at risk of human trafficking were the fact that in some communities, having a close relative who is abroad is perceived as a status symbol, the ineffective law enforcement and inadequate punishment of human trafficking brokers, as well as the poor co-ordination and cooperation among concerned government agencies and communities. Finally, the effects of the identified reasons and potential factors for human trafficking need to be studied quantitatively to measure the influence of each factor on trafficking as well as to facilitate the prioritization of future interventions.

## Data Availability

All relevant data are within the manuscript. The data upon which these findings were developed can also be available upon request.

## Abbreviations

FGD: Focus Group Discussion
IOM: International Organization for Migration
NGO: Nongovernment Organizations
SEM: Social Ecological Model
SNNPRS: Southern Nations Nationalities and Peoples Regional State
